# No Changes in the Occurrence of Methicillin-Resistant *Staphylococcus aureus* (MRSA) in South-East Austria during the COVID-19 Pandemic

**DOI:** 10.3390/pathogens12111308

**Published:** 2023-11-02

**Authors:** Gernot Zarfel, Julia Schmidt, Josefa Luxner, Andrea J. Grisold

**Affiliations:** 1 Diagnostic and Research Center for Molecular BioMedicine, Medical University of Graz, 8010 Graz, Austria; gernot.zarfel@medunigraz.at (G.Z.); julia.schmidt@edu.fh-joanneum.at (J.S.); josefa.luxner@medunigraz.at (J.L.); 2 Biomedical Science, University of Applied Sciences, 8020 Graz, Austria

**Keywords:** MRSA, *spa* typing, ST398, epidemiology, CC127

## Abstract

Methicillin-resistant *Staphylococcus aureus* (MRSA) is a universal threat. Once being well established in the healthcare setting, MRSA has undergone various epidemiological changes. This includes the emergence of more aggressive community-acquired MRSA (CA-MRSA) and the occurrence of MRSA which have their origin in animal breeding, called livestock-associated MRSA (LA-MRSA). Emergence of new clones as well as changes in the occurrence of some clonal lineages also describes the fluctuating dynamic within the MRSA family. There is paucity of data describing the possible impact of the COVID-19 pandemic on the MRSA dynamics. The aim of the study was the analysis of MRSA isolates in a three-year time period, including the pre-COVID-19 years 2018 and 2019 and the first year of the pandemic 2020. The analysis includes prevalence determination, antibiotic susceptibility testing, *spa* typing, and detection of genes encoding the PVL toxin. The MRSA rate remained constant throughout the study period. In terms of a dynamic within the MRSA family, only a few significant changes could be observed, but all except one occurred before the start of the COVID-19 pandemic. In summary, there was no significant impact of the COVID-19 pandemic on MRSA in Austria.

## 1. Introduction

Methicillin-resistant *Staphylococcus aureus* (MRSA) is a multiple-resistant pathogen, responsible for a wide spectrum of diseases, ranging from colonization only to mild skin infections up to life-threatening invasive diseases. Since the first description of methicillin-resistant *Staphylococcus aureus* (MRSA) in 1961 in the UK, several epidemiological changes have been noted [1,2].

Originally, MRSA were limited to the clinical setting. These MRSA, also referred to as hospital acquired (HA)-MRSA, still account for the majority of MRSA strains isolated in mainly older patients with underlying co-morbidities [1]. In the 1980s, new strains of MRSA emerged in people who were not among the classic MRSA risk patients and which tend to be, due to the expression of the Panton-Valentine-Leucocidin (PVL) toxin, more aggressive. These strains, which are mainly found in younger individuals and in the general human population, have been grouped together as community-acquired (CA)-MRSA [3,4,5].

In 90% of the cases, CA-MRSA cause skin and soft-tissue infections, and in contrast to HA-MRSA CA-MRSA, are susceptible to most antibiotics other than ß-lactams. Outbreaks of CA-MRSA have been associated with several common features including crowding, frequent skin-to-skin contact, compromised skin, or shared equipment/supplies [2,3]. The third major group is the animal breeding-associated (LA)-MRSA, discovered around the turn of the millennium [6,7].

In the laboratory, various molecular typing methods are used to assign these different MRSA groups to clonal complexes or to determine their *spa* types in order to map the global distribution of certain MRSA clones [1,2,8].

With the onset of the COVID-19 pandemic, many things changed both in the hospital and in the general population. As MRSA is mainly transmitted via hands, the restrictive hygiene measures in hospitals, but also social distancing, as well as local lockdowns in the general population reduced person-to-person contacts to a minimum and thus certainly also the probability of direct MRSA transmission. In addition to spreading within the healthcare system, MRSA, like other multidrug-resistant pathogens, spread across national borders via travelers or goods. The complete restriction of travel and the international transport of goods might, therefore, have an impact on the spread of pathogens [9,10].

There are still relatively few data for Austria describing the impact of the COVID-19 pandemic on multi-resistant bacteria; in particular, there are no studies on MRSA and its dynamics. Therefore, the aim of this study was to determine the occurrence of MRSA in Austria and its characterization in the pre-COVID-19 years 2018, 2019 and the year 2020.

## 2. Material and Methods

### 2.1. Bacterial Isolates

This study was performed at the Institute of Hygiene, Microbiology and Environmental Medicine, Medical University Graz, Austria. Clinical samples were obtained from the University Hospital of Graz (approximately 1200 beds), from eight peripheral hospitals and from local practitioners in the district Styria, in the South-East of Austria. MRSA isolates from January 2018 to December 2020 were included.

Bacterial identification and antibiotic susceptibility testing were performed by using the semi-automated VITEK II instrument (bioMérieux, Marcy l’Etoile, France). All MRSA primary isolates were routinely stored at −70 °C. For this study, MRSA were retested, and resistance to cefoxitin was confirmed by Etest (AB Biodisk, Solna, Sweden). Resistance to following non-ß-lactams antibiotics was analyzed: ciprofloxacin, clindamycin, daptomycin, erythromycin, fosfomycin, fusidic acid, gentamicin, levofloxacin, linezolid, mupirocin, rifampicin, teicoplanin, tetracycline, tigecycline, trimethoprim-sulfamethoxazole (SXT), vancomycin.

### 2.2. Genetic Analyses

*Spa* typing, DNA purification, and PCR were performed as described previously. In brief: A loopful of material from an overnight culture (grown on blood agar plates) was suspended in 0.5 mL sterile H_2_O. The suspension was heated to 95 °C for 10 min and 1.0 μL of the suspension was used for PCR. The oligonucleotides used for amplification correspond were 1113F, 5′-TGTAAAACGACGGCCAGTTAAAGACGATCCTTCGGTGAGC and (1514R, 5′-CAGGAAACAGCTATGACCCAGCAGTAGTGCCGTTTGCTT. PCR conditions were 95 °C for 5 min; 35 cycles each of 95 °C for 15 s, 58 °C for 30 s, and 72 °C for 45 s; and a final step at 72 °C for 10 min [11]. The *spa* types and appropriate BURP clusters were assigned by using RidomStaphType software (http://www.ridom.de/staphtype, accessed on 1 October 2021).

PCR amplification for the lukF/lukS-PV genes encoding the components of the PVL toxin was performed as described by Lina et al. [12].

### 2.3. Classification of HA-MRSA, CA-MRSA, LA-MRSA

In this study, assignment to the three MRSA groups HA-MRSA, CA-MRSA, and LA-MRSA was based on the known genetic differences as described by Lakhundi et al. [1]. CA-MRSA were all isolates positive for the PVL toxin as the main virulence determinant and causative agent of skin and soft tissue infections. LA-MRSA isolates were isolates with the typical *spa* types CC011 and CC127 and must be PVL-negative. Isolates of types CC011 and CC127 that contained PVL were classified as CA-MRSA. HA-MRSA were all isolates that were not classified as CA- or LA-MRSA.

## 3. Results

Within the three-year study period, a total of 11,114 *Staphylococcus aureus* single-patient isolates were detected (3780/2018, 3926/2019 and 3408/ 2020). From those, the MRSA phenotype was found in 637 isolates, with 220 isolates (5.8%) in 2018, 237 isolates (5.8%) in 2019, and 180 isolates (5.2%) in 2020, respectively. The decrease in MRSA in 2020 was not significant (*p* = 0.28).

### 3.1. Antibiotic Resistance

**Figure 1:**
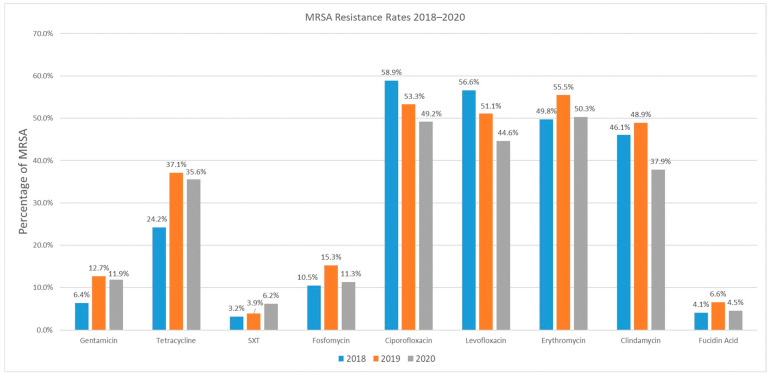
Proportion of resistance rates in MRSA, isolated from 2018–2020. Only the increase in tetracycline-resistant MRSA isolates from 2018 to 2019 (*p* < 0.01) and the decrease of clindamycin in 2020 was significant (*p* = 0.027); all other changes were not significant (SXT = trimethoprim-sulfamethoxazole).

Regarding the occurrence of resistance, all isolates were susceptible to the three antibiotics, vancomycin, linezolid, and tigecycline. Four antibiotics each had a resistant isolate in only one of the years studied: teicoplanin (one isolate in 2018), daptomycin (one isolate in 2018), mupirocin (one isolate in 2020), and rifampicin (in 2019). Gentamicin, fosfomycin, erythromycin, and fusidic acid were found to have consistent resistance rates in each of the years studied (Figure 1). Resistance rates for fluoroquinolones decreased starting from 2018 on, with ciprofloxacin from 58.9% (2018), 53.3% (2019) to 49.2% (2020) and for levofloxacin from 56.6% (2018), 51.1% (2019) to 44.6% (2020). It is considered a continuous process and not triggered by the COVID-19 pandemic (Figure 1).

The highest decrease in resistance rates was found in clindamycin in 2020, with a decrease from 48.9% to 37.9%, which was one of the significant values that could be observed within the study period (*p* = 0.027).

The second significant change in the resistance rates was observed in tetracycline, with an increase from 24.2% in 2018 up to 37.1% in 2019 (*p* < 0.01). Therefore, this change occurred before the start of the COVID-19 pandemic. In 2020, the resistance rate to tetracycline was 35.6%, which is a not significant decrease. For SXT, there was an increase in the resistance rates from 3.2% (2018) and 3.9% (2019) up to 6.2% in 2020, but that was also not significant (Figure 1).

### 3.2. Spa Typing

Looking at the *spa* types, the diversity of *spa* types has increased during the three-year study period. Whereas 65 different *spa* types were found in 2018 (3.4 isolates per *spa* type), in 2019 there were 80 different *spa* types (2.9 isolates per *spa* type), and in 2020, 71 (2.5 isolates per *spa* type).

In total, there were 145 different *spa* types, which could be assigned to 12 different *spa* clonal complexes (CC) (Supplementary Table S1). The most common six *spa* CC, which accounted for more than 80% of all isolates in each year, were *spa*CC002, *spa*CC008, *spa*CC011, *spa*CC021, *spa*CC032, and *spa*CC127 (Table 1).

There were two significant changes; the decrease in *spa*CC032 from 29.9% (2018) to 19.7% (2019) (*p* = 0.02) and the increase in *spa*CC127 from 6.8% (2018) to 14.4% (2019) (*p* = 0.01), both of which occurred prior to the COVID-19 year 2020 (Table 1).

The 637 MRSA detected during the study period were assigned to the following MRSA types.

### 3.3. CA-MRSA

**Figure 2:**
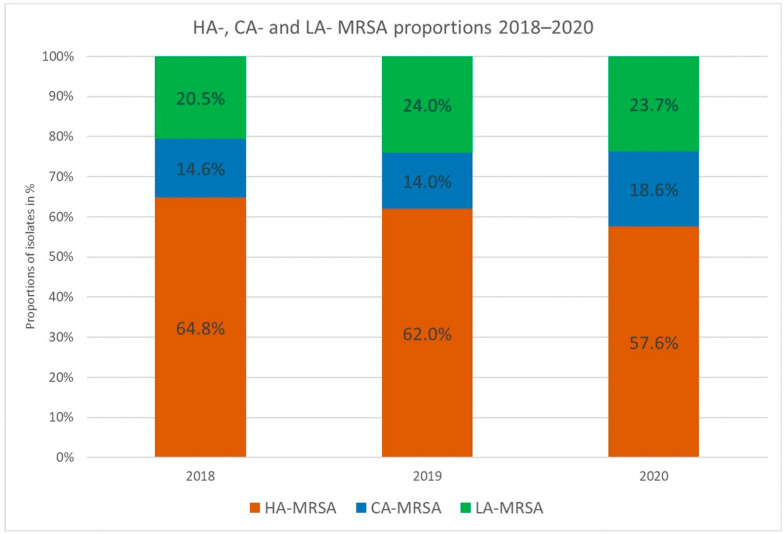
Proportion of MRSA types, isolated from 2018–2020 in Austria. None of the changes incidence of HA-, CA-, or LA-MRSA were significant.

A total of 97 CA-MRSA were found. The overall proportion of PVL toxin-producing CA-MRSA isolates increased from 14.6% (32/219) in 2018 and 14.0% (32/229) in 2019 to 18.6% (33/177) in 2020. However, this increase was not significant (*p* = 0.89 for 2018 to 2019 and *p* = 0.22 for 2019 to 2020) (Figure 2).

### 3.4. LA-MRSA

As for LA-MRSA, 142 isolates were assigned to LA-MRSA; 45 (20.5%) in 2018, 55 (24.0%) in 2019, and 42 (23.7%) in 2020. While the number of LA-MRSA remained stable throughout the study period, the changes in the percentage were always not significant (*p* = 0.43 for 2018 to 2019 and *p* = 1 for 2019 to 2020) (Figure 2).

### 3.5. HA-MRSA

The proportion of HA-MRSA isolates decreased from 142 (64.8%) isolates in 2018 to 142 (62.0%) isolates in 2019, and finally, 102 (57.6%) isolates in 2020. None of the changes were significant (*p* = 0.56 for 2018 to 2019 and *p* = 0.41 for 2019 to 2020) (Figure 2).

## 4. Discussion

With the onset of the COVID-19 pandemic, numerous infection control and prevention measures were taken, including mask wearing, forced hand hygiene, quarantine or social distancing [13,14,15].

While these measures were intended to contain the transmission of SARS-CoV-2, it was the high use of antibiotics in SARS-CoV-2 patients that soon raised the question of what impact the COVID-19 pandemic would have on the emergence and spread of multidrug-resistant pathogens, including MRSA [16,17].

Gram-negative pathogens including carbapenem-resistant *Acinetobacter baumannii* (CRAB), carbapenem-resistant *Pseudomonas aeruginosa* (CRPA), and third-generation cephalosporin-resistant and carbapenem-resistant Enterobacterales (CRE) are listed as critical priority pathogens, followed by priority pathogens including gram-positive bacteria such as Methicillin-resistant *Staphylococcus aureus* (MRSA) and vancomycin-resistant *Enterococcus faecium* [18].

Studies reporting on the impact of the COVID-19 pandemic on the prevalence of multidrug-resistant bacteria do not yet provide a consistent picture. Abukakar et al. showed in a review that changes in multi-resistant bacteria vary depending on the study [19]. While most studies reported an increase in Carbapenem-resistant Enterobacterales (CRE), or Carbapenem-resistant *Acinetobacter baumannii*, there are also some publications showing a reduction of CRE [10,19].

The same was found for MRSA. Just over half (54.5%) of the studies analyzed reported an increase in MRSA prevalence, with this increase varying between 4.6% and 200% [19].

It should be noted that the prevalence of MRSA varies from region to region and was even declining in some countries prior to the COVID-19 pandemic [10,19,20,21,22,23].

Within this study, COVID-19 had no effect on the number of *S. aureus* isolates in our patients nor on the incidence of MRSA isolates, with a MRSA rate of 5.2–5.8%.

The COVID-19 measures do not only focus on hospitals. The management of the pandemic also included a decrease in local and international travel, as well as local lockdowns and quarantine at home. While HA-MRSA occur primarily in the medical sector, and CA-MRSA are found in outpatients without predisposing factors, and also often associated with travel, none of the measures described above affected the agricultural sector [1]. All this raises the question of whether the distribution of the different types of MRSA may have changed.

Even if we could observe a slight decrease in HA-MRSA in this study, this decrease already started in the pre-COVID-19 period. The assignment of LA-MRSA and CA-MRSA can be difficult in some cases, if the assignment is based solely on tetracycline resistance as a characteristic feature of LA-MRSA. In this study, we found some isolates that were resistant to tetracycline but additionally positive for PVL and assigned these isolates to CA-MRSA (PVL beats tetracycline resistance and *spa* type). All in all, the rate of LA-MRSA with nearly a quarter of isolates is remarkable, but is based on the high density of pig farming in our region [1,2,23].

Thus, with a focus on the assignment to HA-, CA-, and LA-MRSA, there were no significant changes within the analyzed years. We found at least changes for individual clonal CC lineages, with a significant increase in the occurrence of CC127 isolates and a decrease in CC032, but changes in the frequency of individual clonal lineages are not unusual in MRSA. Therefore, looking at the first COVID-19 year, we cannot see any influence of the COVID-19 pandemic on MRSA in Austria [23,24].

The vast majority of HA-MRSA strains are, in addition to being resistant to β-lactam antimicrobials, resistant in particular to aminoglycosides, macrolides, lincosamides, and fluoroquinolones; resistance to vancomycin and linezolid, for example, is still rare [19].

This was also the case in this study, with all MRSA isolates being susceptible to vancomycin, linezolid, and tigecycline, and only individual MRSA isolates being resistant to teicoplanin, daptomycin, mupirocin, and rifampicin.

The significant decrease in the resistance rate to clindamycin can certainly be explained by the reduction in lincosamide administration observed in Austria, which decreased from 0.70 defined daily doses (DDD) per 1000 inhabitants per day (2011) to 0.53 DDD per 1000 inhabitants per day in hospitals and from 0.84 DDD per 1000 inhabitants per day (2011) to 0.53 (2020) in outpatients [20].

The significant change in tetracycline resistance, with an increase from 24.2% in 2018 to 37.1% in 2019, can be directly attributed to the increase in CC127, but not COVID-19, as it started before COVID-19 [25].

## 5. Conclusions

Changes in the incidence and distribution of MRSA (HA-, CA-, LA-MRSA) in Austria could not be observed during the both pre-COVID-19 years 2018 and 2019 and the first year of the COVID-19 pandemic.

The few changes that could be observed in this study occurred mostly in the two years before COVID-19 started.

Further analyses and cross-country comparisons will show the long-term effect of the COVID-19 pandemic on the occurrence of multi-resistant bacteria, including MRSA.

## Figures and Tables

**Table 1:** Presence of the six most common *spa* CCs in 2018, 2019, and 2020. The percentage of the total number of isolates in the indicated years and the absolute numbers in parentheses are given.

	*spa*CC002	*spa*CC008	*spa*CC011	*spa*CC021	*spa*CC032	*spa*CC127
2018	26.5% (58)	10.0% (22)	12.8% (28)	4.6% (10)	29.7% (65)	6.8% (15)
2019	24.9% (57)	9.6% (22)	14.0% (32)	2.2% (5)	19.7% (45)	14.4% (33)
2020	18.6% (33)	11.9% (21)	15.3% (27)	4.0% (7)	20.3% (36)	14.1% (25)
2018–2020	23.7% (148)	10.4% (65)	13.9% (87)	3.5% (22)	23.4% (146)	11.7% (73)

## Data Availability

Data is contained within the article or Supplementary Materials.

## Supplementary Materials

The following supporting information can be downloaded at: https://www.mdpi.com/article/10.3390/pathogens12111308/s1, Table S1: List of all MRSA isolates including their respective *spa* types, PVL-toxin, and resistance pattern.
